# Supplementary material for: All-at-once RNA folding with 3D motif prediction framed by evolutionary information
Source: Nat Methods. 2025 Oct 3;22(10):2094–106. doi: 10.1038/s41592-025-02833-w (PMC12510878; doi:10.1038/s41592-025-02833-w)
Supplement: Supplementary file 3 — Source code to run CaCoFold-R3D. Data and results. [file 41592_2025_2833_MOESM3_ESM.gz › supplemental_material/results/CaCoFold-R3D/CaCoFold-R3D_Rfam_vshuffle/RF01956_NEAT1_2.R2R.sto.pdf]

RF01956_NEAT1_2

5′-C C C U U U G G A C U U U ● ○ C Y C U A G G U U C ● G U G C U A A ● C U C U U C U G U G A G C U C A C U C U R C C C ○ U Y C U C C U C C C U U U A A C U Y U ● ○ ○ R A G G U U A U Y ● ● U U C A C U U A A A A C ● U U G C Y U C U G G U
